# Identification of Novel Molecular Therapeutic Targets and Their Potential Prognostic Biomarkers Based on Cytolytic Activity in Skin Cutaneous Melanoma

**DOI:** 10.3389/fonc.2022.844666

**Published:** 2022-03-08

**Authors:** Haoxue Zhang, Yuyao Liu, Delin Hu, Shengxiu Liu

**Affiliations:** ^1^ Department of Dermatovenerology, The First Affiliated Hospital of Anhui Medical University, Hefei, China; ^2^ Key Laboratory of Dermatology, Ministry of Education, Hefei, China; ^3^ Inflammation and Immune Mediated Diseases Laboratory of Anhui Province, Anhui Medical University, Hefei, China; ^4^ Department of Burns, The First Affiliated Hospital of Anhui Medical University, Hefei, China

**Keywords:** skin cutaneous melanoma, cytolytic activity, genes, prognosis, therapies, clinical guidelines

## Abstract

Skin cutaneous melanoma (SKCM) attracts attention worldwide for its extremely high malignancy. A novel term cytolytic activity (CYT) has been introduced as a potential immunotherapy biomarker associated with counter-regulatory immune responses and enhanced prognosis in tumors. In this study, we extracted all datasets of SKCM patients, namely, RNA sequencing data and clinical information from The Cancer Genome Atlas (TCGA) database and the Gene Expression Omnibus (GEO) database, conducted differential expression analysis to yield 864 differentially expressed genes (DEGs) characteristic of CYT and used non-negative matrix factorization (NMF) method to classify molecular subtypes of SKCM patients. Among all genes, 14 hub genes closely related to prognosis for SKCM were finally screen out. Based on these genes, we constructed a 14-gene prognostic risk model and its robustness and strong predictive performance were further validated. Subsequently, the underlying mechanisms in tumor pathogenesis and prognosis have been defined from a number of perspectives, namely, tumor mutation burden (TMB), copy number variation (CNV), tumor microenvironment (TME), infiltrating immune cells, gene set enrichment analysis (GSEA) and immune checkpoint inhibitors (ICIs). Furthermore, combined with GTEx database and HPA database, the expression of genes in the model was verified at the transcriptional level and protein level, and the relative importance of genes in the model was described by random forest algorithm. In addition, the model was used to predict the difference in sensitivity of SKCM patients to chemotherapy and immunotherapy. Finally, a nomogram was constructed to better aid clinical diagnosis.

## Introduction

Skin cutaneous melanoma (SKCM) is one of the most lethiferous malignancies. Though SKCM only constitutes ~5% of all skin cancers, it accounts for >75% of skin cancer deaths (1). Currently, most melanomas are removed *via* the standard surgical technique that excises both the tumor and a margin of normal appearing skin (2). Unfortunately, surgical resection offers so little in the management of individuals with regional or distant metastases (3). Adjuvant therapies, such as radiotherapy, immunotherapy, biochemotherapy, can possibly benefit postoperative patients (4). But the conventional treatments have not improved the outcomes of SKCM, which may be due to the hypo-responsiveness and inherent resistance of melanoma cells (5). Immunotherapy has promised an optimizing future for SKCM in recent years (6–8), managing to enhance the prognosis of SKCM patients. Though it has shown great clinical effect, only a small percentage of patients profit by long-range treatment (9). Many factors like the tumor types (10), and age (11) have potential influence on the efficacy. Therefore, establishment of an efficient prognosis model is essential, and it can direct clinical treatment of SKCM patients.

Immune checkpoints refer to a plethora of inhibitory pathways hardwired into the immune system that are crucial for maintaining self-tolerance and regulating the strength of the peripheric immune system to minimize collateral tissue damage, realizing immune evasion in tumors (12). Therefore, immune checkpoint inhibitors (ICIs) are emerging as a promising antitumor immunotherapy. ICIs are able to unleash anti-tumor immunity and mediate durable cancer regressions (13) *via* inhibition of pathways like the cytotoxic T-lymphocyte-associated protein 4 (CTLA-4), programmed cell death-1 (PD-1), and programmed cell death ligand-1 (PD-L1). Elevated evidences have substantiated the use of ICIs in SKCM (14), starting with the earliest approval of an anti-CTLA-4 drug called ipilimumab for advanced-stage melanoma in 2011 (15). Currently, pembrolizumab and nivolumab, both inhibitors of PD-1, also are popularly used in clinical. Combination ICI therapy has shown unprecedented, long-lasting survival benefits in the treatment of metastatic melanoma (16). However, despite the impressive effects, a large proportion of patients do not respond to these drugs. A key challenge is to understand the variability of immune responses to ICIs. Granule exocytosis (perforin and granzymes) is considered as one of main pathways involved in cytotoxic lymphocyte-mediated tumor cell death, and it plays a crucial role in killing cancer cells during cancer immunosurveillance and immunotherapy (17). Michael et al. innovatively designed the cytolytic activity (CYT) score based on expression levels of granzyme A (GZMA) and perforin (PRF1) that relates with immune responses to ICIs immunotherapies and predicts prognosis (18). Zaravinos et al. once investigated that the CYT-high subgroup in colorectal cancer can be benefited to a higher percentage from ICIs immunotherapies (19). So, it is potentially valuable to explore genes related to CYT and define its ultimate effect.

Thus, on the whole, in this article, we probed the RNA sequence data from 446 SKCM specimens to find that CYT was a valuable prognostic biomarker for patients with SKCM. We also discovered that CYT may regulate tumor mechanism in many ways, which provides new ideas for the immunotherapy on SKCM.

## Materials and Methods

### Collection of SKCM Samples and Datasets

As conducting this research, several datasets from public databases were used. We downloaded the HTSeq-FPKM gene expression data and corresponding clinical information of all SKCM patients from the official website of the TCGA database (https://www.cancer.gov). We collected 472 samples in total (namely, one normal tissue sample and 471 SKCM tissue samples). Cases with incomplete clinical data were excluded. Finally, a total of 446 patients with full follow-up information were enrolled. In the process of further validation, we employed GSE65904 and GSE54467 matrices from the public repository of the Gene Expression Omnibu (GEO) (https://www.ncbi.nlm.nih.gov/geo/).

### Evaluation of the Prognostic Value of CYT

In order to clearly define the prognostic value of CYT in SKCM, we performed KM survival analysis (an event dependent analyzing form to provide more accurate measurement of survival rates at different intervals (20)) and univariate Cox regression analysis on the overall survival (OS), disease specific survival (DSS), and progression-free-survival (PFS) of patients in the TCGA-SKCM dataset. We also combined results derived from the univariate Cox regression analysis of GSE65904 and GSE54467 to conduct a meta-analysis.

### Identification of CYT-Related Genes (CYTRG) and Prognosis-Related CYTRG

Patients in the TCGA-SKCM dataset were grouped into a high-CYT and a low-CYT group by median split, and then we used differential analysis on both groups in order to identify genes that could characterize CYT that ‘CYTRG’. Prognosis-correlated CYTRG for SKCM patients were then recognized using univariate Cox regression analysis on CYTRG and corresponding clinical data.

### Identification of Subgroups and Evaluation of Subgroups

Then non-negative matrix factorization (NMF) clustering was applied on the CYTRG to classify new subgroups (clusters 1 and 2) of SKCM patients using the NMF R package. NMF is widely used in bioinformatics and with its ability to extract meaningful information from high-dimensional data (21), the use value of identified CYTRG was accordingly confirmed. We conducted KM survival analysis, compared number of somatic mutations and performed Gene Set Variation Analysis (GSVA) to determine the discrimination between C1 and C2 groups. The Estimation of Stromal and Immune cells in Malignant Tumor tissues using Expression data (ESTIMATE) algorithm was used to calculate stromal score, immune score, and ESTIMATE score of the different subgroups. The abundance of tumor-infiltrating immune cells in the different subpopulations was then assessed using the Microenvironment Cell Populations-counter (MCP-counter) method, which was introduced by Becht et al. (22) that allows the robust quantification of the absolute abundance of eight immune and two stromal cell populations in heterogeneous tissues from transcriptomic data.

### Establishment of the CYT−Related Prognostic Model

A total of 446 representative patients were extracted from the TCGA repository. They possessed complete survival information and all relevant clinical features, such as age, sex, tumor stage and tumor-node-metastasis (TNM) stage. We employed lasso-cox regression analysis to screen out crucial CYTRG that have close relation with DSS. Certain CYT-related coefficients (βi) were calculated with the multivariate Cox regression model. The risk score formula (Expi) that was composed of βi and expression levels of CYTRG was set up. The equation ‘Risk score = ∑ (β_i_ ∗ Exp_i_)’ was used to calculate each risk score for every patient. The samples were classified into either a high-risk or a low-risk cohort according to the cut-of (based on the median risk score). Using R software (version 4.04), KM survival analysis and log-rank test were performed to compare DSS in either high-risk or low-risk group.

### Evaluation of This Prognostic Model

Then, a receiver operating characteristic (ROC) curve was generated by the R package survival ROC (23) and was used to understand the diagnostic value of this model (24). Also, we adopted the calibration curve method (CCM), principal component analysis (PCA), decision curve analysis (DCA) to further estimate the accuracy of this prognostic model. We evaluated the prognostic significance of the risk scores and also clinical variables, like age, sex, TNM staging, *via* univariate and multivariate Cox regression analyses. Moreover, according to the results from multivariate Cox regression analysis combined with tumor mutation burden (TMB), a nomogram was then built and concurrently could be used to predict DSS for the 1-year, 3-year, and 5-year of each SKCM patient. Briefly speaking, TMB refers to the number of mutations that exist within a tumor, and high TMB values are observed in melanoma and have been thought to be associated with responses to ICIs (25). The prognostic value of the novel model and the characteristic nomogram was further compared with the tumor staging system, TMB, age, tumor purity and gender in terms of the DCA plots, concordance index (Cindex), and restricted mean survival (RMS) curves.

### Drug Sensitivity Analysis

Since chemotherapy is commonly applied to treat SKCM, we utilized R package “pRRophetic” to assess the chemotherapeutic response determined by the half maximal inhibitory concentration (IC50) of each SKCM patient on the Genomics of Drug Sensitivity in Cancer (GDSC) website. Besides, to elucidate the effects of CYT-related genes on drug sensitivity and tolerance in this model, we acquired transcriptome data from the CellMiner database (https://discover.nci.nih.gov/cellminer/) and FDA-certified drug sensitivity-related data. Then we utilized a Pearson correlation test to analyze the relationship between gene expression and drug sensitivity. The programmed cell death 1 (PDCD-1, also known as PD-1) and cytotoxic T-lymphocyte associated protein 4 (CTLA-4) pathways have been implicated in tumor immune evasion. So immune checkpoint inhibitors targeting PD-1 and CTLA-4 may thereby improve antitumor immunity. The immunophenoscore (IPS) was used to predict clinical responses to immune checkpoint inhibitors (26). The data of the IPS in SKCM patients were download from the Cancer Immunome Database (TCIA) (https://tcia.at/home). These results are able to better guide doctors in choosing different drug treatment on patients.

### Expression and Modulation of Genes in the Signature

We conducted differential analysis on expression levels of genes in the signature between normal samples and tumor samples. We then searched for differential expression of genes between the high-risk and low-risk groups. The Human Protein Atlas (HPA) database (http://www.proteinatlas.org) was generated by Uhlén et al. (27), and contains an invaluable resource of human protein-coding genes, enlightening researchers on gaining insights of human proteins. Thus we explored the expression of CYTRGs represented in this signature in normal skins and SKCM tissues using the HPA database. The expression of one certain gene was investigated in normal and cancer tissues using the same antibody. Then we conducted spearman correlation analysis to demonstrate the relationship between CYT and genes in our model, which helped to confirm the rationality of CYTRG identified *via* the differential analysis.

### Mutation Analysis and Tumor Mutation Burden (TMB) Calculation

Mutation analysis was conducted based on all available somatic mutation data of patients from the TCGA cohort. Then we visualized the somatic mutation data in the Mutation Annotation Format (MAF) using the “maftoools” R package, which is efficient and comprehensive and provides various functions for cancer genomic analyses (28). Subsequently, tumor mutation burden (TMB) differential analysis was performed between wild and mutation types based on defined genes in the model. We also conducted differential analysis on TMB between the high-risk and low-risk groups, and combined with TMB, we conducted survival analysis between the two groups.

### Tumor Microenvironment (TME) Analysis

The newly described algorithm, ESTIMATE (Estimation of Stromal and Immune cells in Malignant Tumor tissues using Expression data) method, applied for assessment of the presence of stromal cells and the infiltration of immune cells in tumor samples using gene expression data (29), was used to calculate interstitial score, immune score, ESTIMATE score, and tumor purity for different molecular subpopulations.

### Immune Cell Infiltration, Immune Checkpoint Gene and CYT Analyses

To better clarify the relationship between the tumor immune cell infiltration status and calculated risk scores, 7 software programs, namely, XCELL, TIMER, QUANTISEQ, MCP-counter, EPIC, CIBERSORT-ABS, and CIBERSORT were used to analyze the immune cell infiltration landscape. The lollipop diagram was displayed to show the correlation between risk score and immune infiltrated cells *via* Spearman correlation method. The differences of immune cell content in high-risk and low-risk groups were shown as boxplots using Wilcoxon signed-rank test. Besides, we conducted differential analysis on the mRNA expression of immune checkpoint genes and CYT elements (GZMA and PRF1). We also performed Spearman correlation analysis on PD-1, PD-L1, CTLA-4, CYT, GZMA, PRF1 and calculated risk scores. Furthermore, we ran a correlation analysis between CYT expression and immune cell contents. All results further substantiated the utility value of our signature.

### Gene Set Enrichment Analysis

A Gene Set Enrichment Analysis (GSEA) on risk genes was performed to obtain the GO and KEGG pathways of this model. The gene set enrichment study was conducted to that are expressed between the high and low-risk classes of the MsigDB (c2.cp.kegg.v7.4.symbols.gmt;c5.go.v7.4.symbols.gmt). The gene set permutations were tested 1,000 times to demonstrate its ability to function consistently. The phenotype label was used to forecast adverse events.

### Prediction of the Possibility That SKCM Patients are Grouped as High Risk

After determining which clinical trait has significant difference, a nomogram was drawn to predict whether a patient with SKCM belongs to the high-risk group. Pathological stage and tumor-bearing state are needed to help doctors better utilize this prognostic model.

### Statistical Analysis

All statistical analysis was accomplished by R version 4.0.4 (Institute for Statistics and Mathematics, Vienna, Austria; https://www.r-project.org). The correlation was determined by Spearman correlation analysis. Wilcoxon test and t-test were utilized to compare clinical variables. Survival status was assessed by the Cox regression analysis. OS, DSS and PFS were generated by the Kaplan–Meier method and evaluated by the log- rank test. Two- tailed p <0.05 was considered statistically significant. The sensitivity and specificity of the model were evaluated using ROC curves. Additionally, we verified the confidence of the model using test datasets and entire datasets. Reasonably, hazard ratios (HRs) and 95% confidence intervals (CIs) were used to describe the relative risk.

## Results

### Patients With High CYT Have Better Prognosis

**Figure 1:**
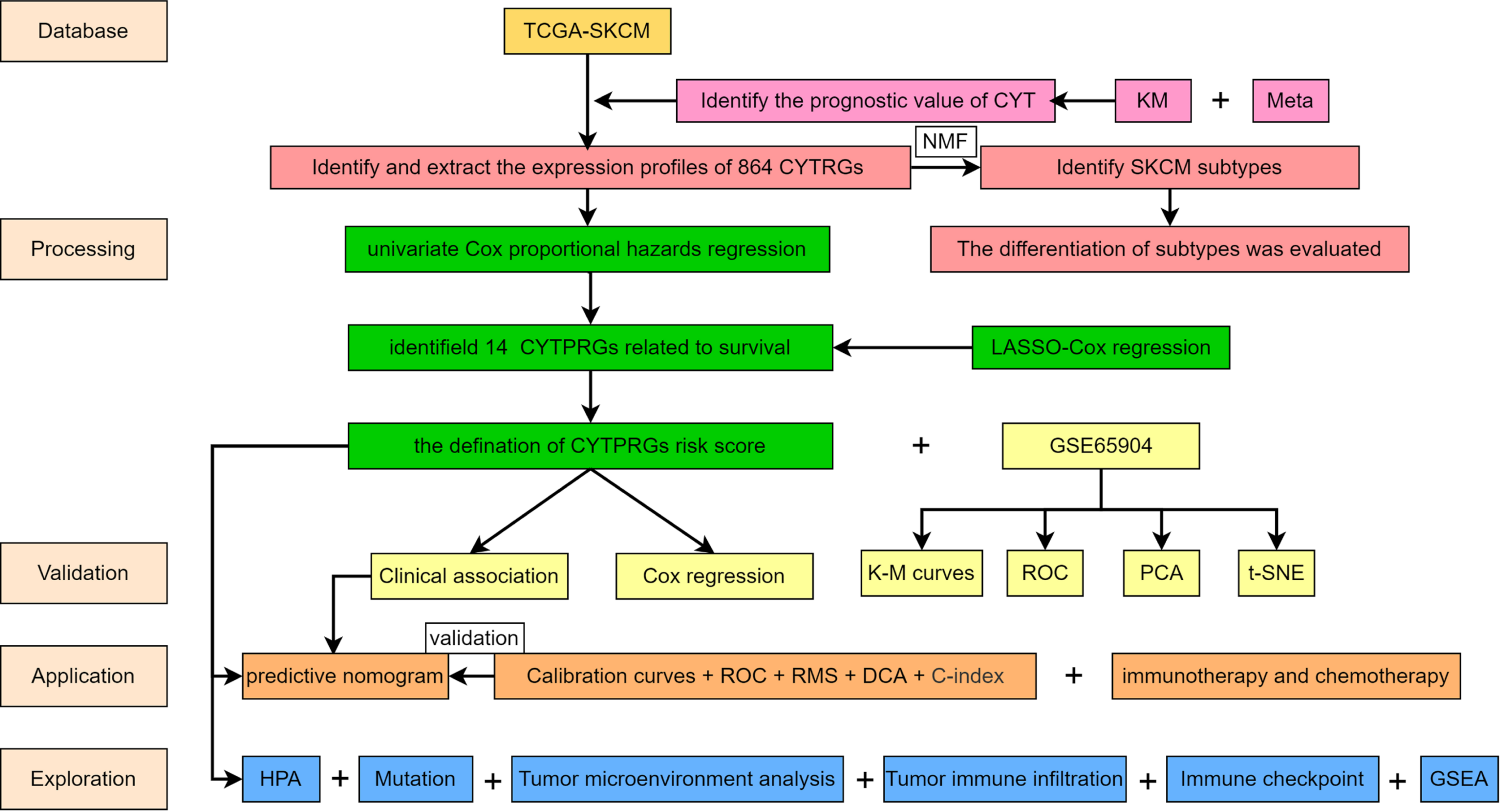
Flow chart of this study.

**Figure 2:**
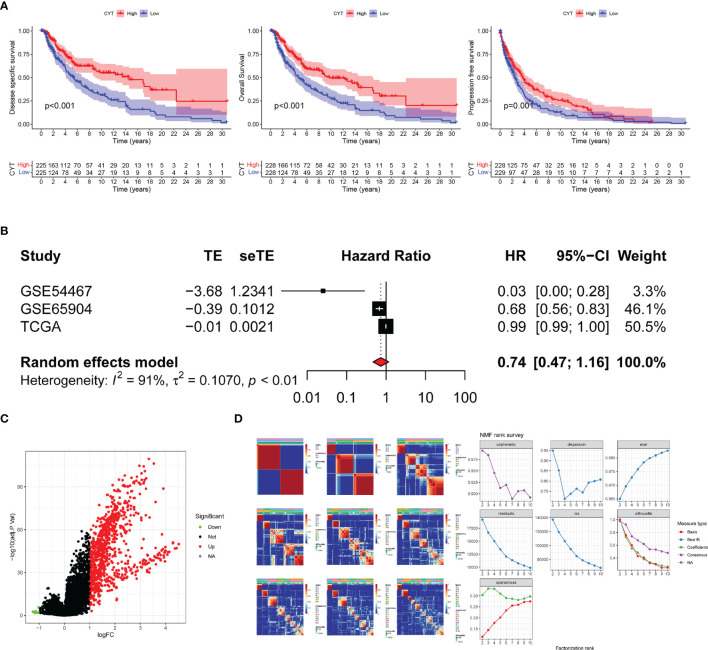
Survival analysis and Meta-analysis. **(A)** Based on values of disease specific survival (DSS), overall survival (OS) and progression-free-survival (PFS), the survival analysis was conducted and the results showed that patients with high-CYT had better prognosis. **(B)** The univariate Cox regression analysis of GSE65904, GSE54467 and the TCGA-SKCM datasets were used to conduct a meta-analysis, which showed that CYT can be a protective factor for SKCM patients with a high heterogeneity, so CYT cannot be used to predict prognosis for SKCM patients directly. The volcano plot displays 864 differentially expressed genes (DEGs) between the high-CYT and low-CYT groups in the TCGA-SKCM cohort **(C)**. Nonnegative matrix factorization (NMF) clustering was conducted and two subgroups were identified the optimal value for consensus clustering **(D)**.

The study design flowchart is shown in **Figure 1**. In total, 471 SKCM tissues and 1 para-cancer tissue were obtained from the TCGA database. After initial screening, 446 samples with full clinical information were included in our study. Detailed clinical features of the samples are shown in **Table 1**. According to the median value of CYT, we separated all SKCM patients into a high-CYT and a low-CYT group, in which we conducted KM survival analysis, and the results indicated that the high-CYT group had better prognosis. Univariate Cox regression analysis told us that CYT was a protective factor validated in 3 independent datasets, and consequently the conclusion came that the higher CYT, the better prognosis for SKCM patients (**Figure 2A**). However, meta-analysis showed that significant heterogeneity remained when CYT was used to predict the prognosis for SKCM patients (**Figure 2B**). Therefore, to enhance prognosis judgment for SKCM, we performed differential analysis respectively on the high-CYT and low-CYT groups, and finally 864 genes that could manifest features of CYT (CYTRG) were identified (**Figure 2C**), which adequately indicated the exploring value of CYT.

**Table 1:** Baseline data of SKCM patients from TCGA cohort.

Covariates	Type	Total	High-risk group	Low-risk group
Age	≤50	139	61	78
	>50	307	162	145
Sex	male	274	146	128
	female	172	77	95
Stage	Stage I	74	26	48
	Stage II	139	91	48
	Stage III	166	76	90
	Stage IV	22	11	11
	unknown	45	19	26
T	T0	23	3	20
	T1	40	14	36
	T2	75	33	42
	T3	87	43	44
	T4	150	99	51
	unknown	71	31	40
M	M0	397	198	199
	M1	23	12	11
	unknown	26	13	13
N	N0	220	115	105
	N1	71	34	37
	N2	49	22	27
	N3	53	25	28
	unknown	53	27	26

### Demonstrating the Value in the Identified CYT-Related Genes (CYTRG)

**Figure 3:**
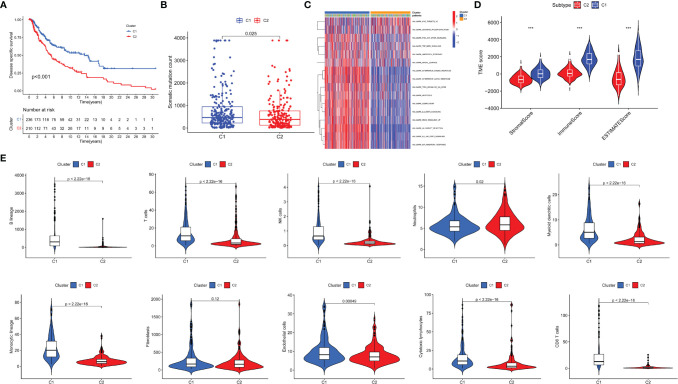
Evaluation of the two newly identified subgroups in terms of their differentiation. Survival analysis **(A)**, Mutation analysis of somatic cells **(B)** and GSEA pathway differential analysis **(C)** on two subgroups. TME analysis of two identified subgroups was conducted **(D)**. The abundance of tumor-infiltrating immune cells was evaluated by MCP-counter and the differential analysis was then conducted **(E)**. *P < 0.05; ***P < 0.001.

To verify the high value of CYT-related genes (CYTRG) for research, we applied non-negative matrix factorization (NMF) clustering method based on the 864 identified genes, and an elementary classification of patient subgroups was set through the NMF consensus clustering, eventually with two subgroups (C1 group, C2 group) sorted out (**Figure 2D**). As shown in **Figure 3A**, the DSS time of each patient in clusters 1 and 2 were visualized and the number of patients at risk was also categorized in two lines. The results showed that patients in C1 group have better prognosis than those in C2 group. Additionally, the somatic mutation count in C1 group was also higher than that in C2 group (**Figure 3B**). The GSVA pathways in C1 group and C2 group showed significant difference too (**Figure 3C**). As shown in **Figure 3D**, the SKCM tissues in cluster 1 showed higher stromal score, immune score, and ESTIMATE score than cluster 2. Also, as shown in **Figure 3E**, the Microenvironment Cell Populations-counter (MCP-counter) algorithm was applied to calculate the abundance of immune cells in SKCM tissues, namely, B cells, T cells, NK cells, Neutrophils, Myeloid dendritic cells, Monocytic lineage, Fibroblasts, Endothelial cells, Cytotoxic lymphocytes, CD8^+^ T cells, with statistically higher abundance of 9 kinds among them in c1 (Neutrophils excluded).

### Establishment and Evaluation of CYT-Based Prognostic Model

**Figure 4:**
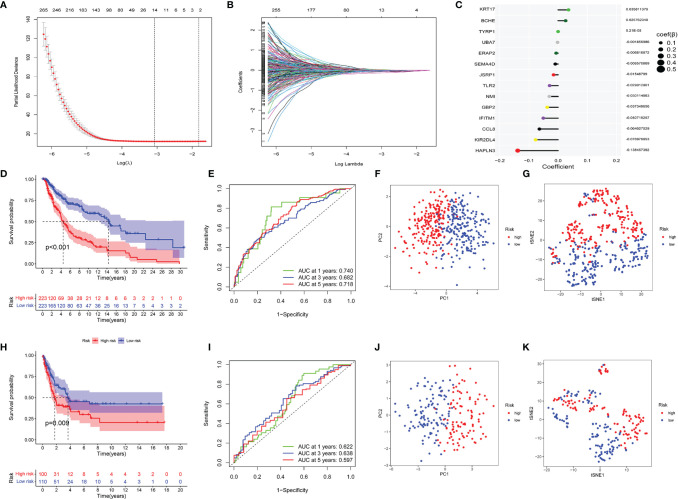
Construction of the CYT-related risk model by Lasso–Cox regression analysis. **(A)** Partial likelihood deviance of variables revealed by the Lasso regression model. The red dots represented the partial likelihood of deviance values, the gray lines represented the standard error (SE), the two vertical dotted lines on the left and right represented optimal values by minimum criteria and 1−SE criteria, respectively. **(B)** Coefficient profiles of the 553 prognosis related CYT-related genes *via* Lasso–Cox regression analysis. **(C)** The coefficient values of 14 key CYT-related genes which were used to build the risk model were listed. Then validating the model. **(D)** Survival analysis, **(E)** ROC analysis, **(F)** Principal component analysis, **(G)** t-SNE analysis of two risk groups of the 14-gene signature in training cohorts, and **(H–K)** in testing cohorts.

In the training sets, univariate Cox regression was used on CYTRG to ascertain 553 prognosis-related CYTRG. Then LASSO-Cox regression analysis was conducted and 14 key CYTRG were screened out (**Figures 4A, B**). β_i_ was calculated using the formula below to establish the risk score model:


$$\text{Risk score}\;=\;\sum (\beta_{i}*{\mathrm{Exp}}_{\text{i}}).$$

This formula was visualized in **Figure 4C**. We set the median score of risk scores as the critical value, and divided 446 patients into the high-risk and low-risk group.

Kaplan–Meier curve showed the DSS of the low-risk group was much better than that of the high-risk group (p <0.001) (**Figure 4D**). ROC had satisfactory sensitivity and specificity (**Figure 4E**). PCA (**Figure 4F**) and t-SNE (**Figure 4G**) indicated high discriminatory power of our model. We obtained similar results using the same methods on the testing sets (**Figures 4H–K**).

**Figure 5:**
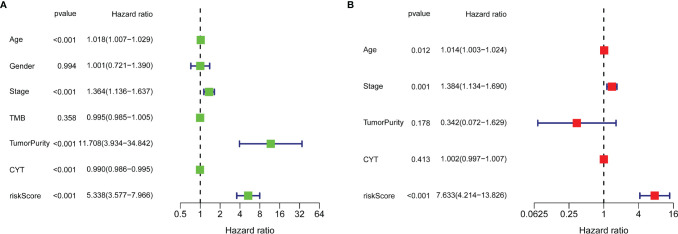
Univariate Cox regression analysis **(A)** and multivariate Cox regression analysis **(B)** illustrated that the 14-gene signature could be used as an independent prognostic factor for SKCM patients.

**Figure 6:**
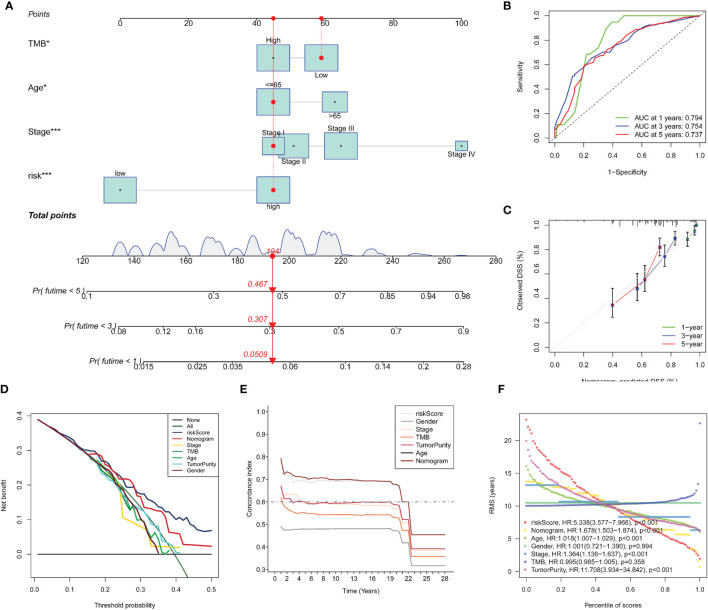
Construction and evaluation of Nomogram. A nomogram constructed by TMB and multi-Cox regression analysis on risk, TNM stage, and age to apply the 14-gene signature in clinical practice **(A)**. ROC curves **(B)** and calibration curves **(C)** indicate that the nomogram is accurate and specific. Further validation of the prognositic value in our signature **(D–F)**. DCA curves for the signature, the nomogram and other clinical traits in terms of their net benefits for SKCM patients **(D)**. Time dependent C-index curves of the model, the nomogram and other clinical traits **(E)**. RMS curves for the signature, the nomogram and other clinical traits and the model has the best potency in predicting prognosis of SKCM patients **(F)**.

Univariate Cox regression analysis (**Figure 5A**) illustrated that indexes CYT, tumor purity, risk score, age and tumor stage were closely associated with DSS. We further performed multivariate Cox analysis (**Figure 5B**), and found that the 14-gene signature could be served as an independent prognostic factor for SKCM (p <0.001), which meant that this signature can be useful to well complement traditional forms of tumor staging. Then we drew a nomogram for model visualization and clinical application, namely, age, tumor stage, TMB and risk score (**Figure 6A**). The area under the curve (AUC) values for the 1-, 3-, and 5-year DSS were “0.794”, “0.754” and “0.737”, predicted by this model (**Figure 6B**). The calibration curve of this predictive model suggested that the model had excellent predictive property and could definitely benefit patients because it exhibited an applicable prediction between the ideal prediction and actual observations (**Figure 6C**). Finally, we used DCA curves, C-index, RMS curves to confirm that this model and the newly-composite nomogram were admissible. The DCA curves showed the comparisons between the clinical net benefit of our model and the nomogram and that of other clinical traits (Staging, TMB, age, tumor putiry, gender) for SKCM patients (**Figure 6D**). Larger net benefits indicated that the model had the excellent clinical effectiveness for bringing benefits for SKCM patients. The C-index of the model and the nomogram was compared with that of other clinical traits, as shown in **Figure 6E**, and the concrete numbers were nearby 0.7, which meant the model was of very moderate to quite important magnitude. RMS curves were recommended by Eng et al. (30) as a flexible and interpretable descriptive technique to represent prognostic biomarkers. As shown in **Figure 6F**, the RMS represents the life expectancy at 20 years (240 months) for SKCM patients with different risk scores. The curve of the model achieved the highest leading position (HR: 5.338; P <0.001), indicating the high precision of our 14-gene signature. On the whole, our results validated the accuracy and feasibility of the signature.

### Immunotherapeutic and Chemotherapeutic Responses of High- and Low-Risk Patients With SKCM

**Figure 7:**
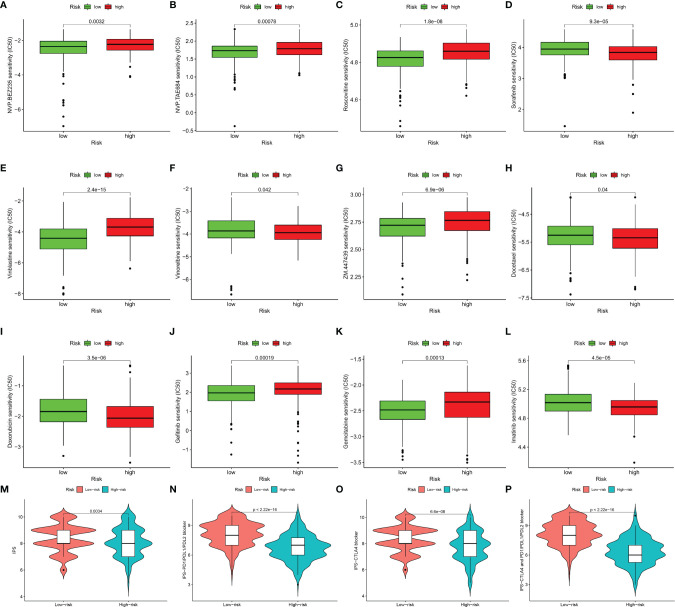
Immunotherapeutic and chemotherapeutic responses high- and low-risk patients with SKCM were shown. Lower IC50 of NVP.BEZ235 **(A)**, NVP.TAE684 **(B)**, roscovitine **(C)**, vinblastine **(E)**, ZM.447439 **(G)**, gefitinib **(J)**, gemcitabine **(K)** were associated with a lower risk score. Lower IC50 of sorafenib **(D)**, vinorelbine **(F)**, docetaxel **(H)**, doxorubicin **(I)**, and imatinib **(L)** were associated with a higher risk score. Distribution of immunophenoscore (IPS) in high-risk *versus* low-risk SKCM subtypes. Violinplot representation of IPS in the high-risk *versus* low-risk groups in CTLA4 negative and PD1 negative group **(M)**, CTLA4 positive and PD1 negative group **(N)**, CTLA4 negative and PD1 positve group **(O)**, and CTLA4 positive and PD1 positive group **(P)**.

Immunotherapy has become a pillar of cancer therapy (31). By far the most widely used immunotherapeutic agents are blocking antibodies targeted to immune inhibitory receptors such as CTLA-4, PD-1, and PD-L1 (15). Unfortunately, not all types of cancer respond to it and not all patients can benefit from it. A lot of research show that strategies that combine traditional chemotherapy and burgeoning immunotherapy synergistically improve the outcome of cancer treatment (32). Expression levels of genes identified in this signature were significantly correlated with the sensitivity of various kinds of drugs by analyzing drug responses in the CellMiner database (**Supplementary Figure S1**). Thus, we further estimated the clinical response to immune checkpoint blockade (targeting CTLA-4 and PD-1 in high- and low-risk patients with SKCM). Then we used R package “pRRophetic” on Genomics of Drug Sensitivity in Cancer (GDSC) (https://www.cancerrxgene.org/) to estimate the half maximum inhibitory concentration (IC50) of chemotherapy response in each SKCM patient (**Figures 7A–L**). Results showed that in high-risk group, more promise in response to sorafenib and imatinib were presented, while gefitinib behaved better in low-risk group. We also investigated the response to chemotherapy in high-risk and low-risk patients with SKCM, and found that 9 chemotherapeutic drugs demonstrated obvious differences in estimated IC50 between high-risk and low-risk groups. Among them, 6 categories (gemcitabine, ZM.447439, NVP.BEZ235, roscovitine, NVP.TAE684 and vinblastine) showed increased sensitivity in low-risk group and the rest 3 categories (vinorelbine, docetaxel and doxorubicin) were more susceptive in high-risk group. In addition, IPS grade analysis showed that the IPS grade among low-risk patients was higher, which meant a better immunotherapy effect (**Figures 7M–P**). These results can better guide drug selection of patients and bring benefit for them.

### Verification the Expression of Genes in the Signature

**Figure 8:**
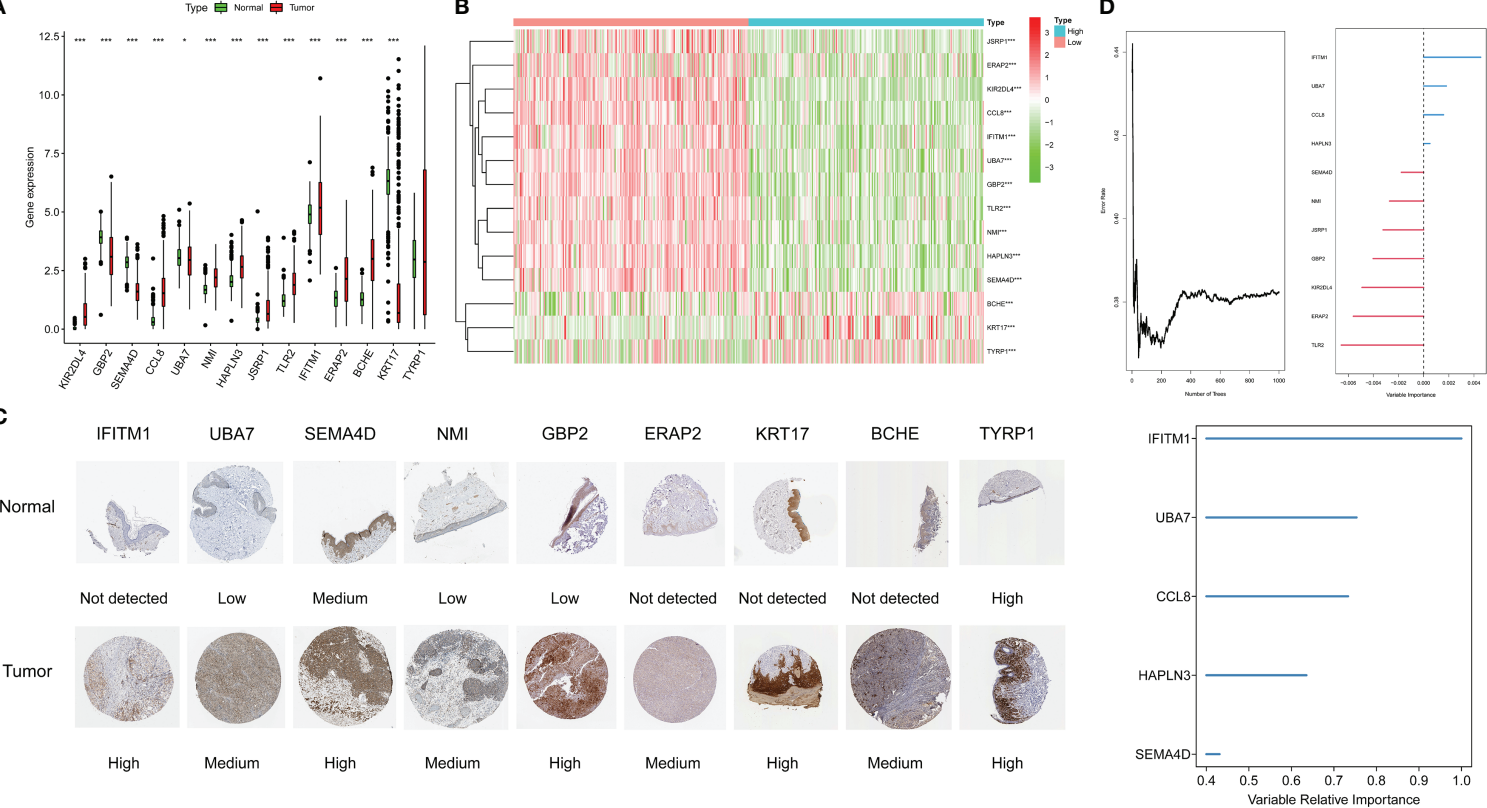
The expression of genes in the signature, the boxplot shows the comparisons between normal types and tumor types **(A)**, the heatmap shows the comparisons between the high-risk and low-risk groups **(B)**, and the immunohistochemical stainings shows 9 gene expression on protein level **(C)**. Error rate for the data as a function of the classification tree, out-of-bag importance values for the predictors **(D)**.

In the boxplot (**Figure 8A**), different expression levels of CYTRG in the signature between normal samples and tumor samples are shown. The heatmap shows the same comparisons between high-risk and low-risk groups (**Figure 8B**). Moreover, based on the HPA database, we intended to make a further validation of CYTRGs in this signature, and stepped forward to potentially confirm the value of these CYTRGs. These 9 recognized characteristic genes (*IFITM1*, *UBA7*, *SEMA4D*, *NMI*, *GBP2*, *ERAP2*, *KRT17*, *BCHE*, and *TYRP1*) (**Figure 8C**) from our model were present in the HPA database, whose differential expression levels between normal skin samples and SKCM samples were consistent with its transcriptional levels in both cohorts, which convincingly supported our findings herein. All immunohistochemical (IHC) images were downloaded from the HPA database. Furthermore, we identified genes with a relative importance >0.4 as the final filtration to highlight the most critical genes. **Figure 8D** shows the relationship between the error rate and the number of classification trees, and it also shows the top five important genes (*IFITM1*, *UBA7*, *CCL8*, *HAPLN3*, and *SEMA4D*). The value of genes in our model was confirmed again from the perspective of gene expression. Promisingly, these results can possibly inspire the scientists to explore CYT-related genes in preventing and curing the disease. The expression levels of CYT were strongly correlated with *KIR2DL4*, *GBP2*, *SEMA4D*, *CCL8*, *UBA7*, *NMI*, *HAPLN3*, *JSRP1*, *TLR2*, and *IFITM1* (cor >0.5), moderately correlated with the expression levels of *ERAP2* (cor >0.3), and weakly correlated with the expression levels of *BCHE*, *KRT17*, and *TYRP1*, which further verified the rationality of differential analysis to identify CYTRG (**Supplementary Figure S2**).

### Calculation of Mutations of Somatic Cells in SKCM Patients

The landscape of mutations of 14 hub genes in the signature was shown in the waterfall map (**Supplementary Figure S3A**). The *KIR2DL4* gene nourished the highest frequency of nonsynonymous mutation in SKCM patients. The bulk mutation type of 13 genes is missense mutation, only *ERAP2* gene has the most frequent mutation type as nonsense mutation. The boxplot displays the TMB difference of each gene in TCGA-cohort (**Supplementary Figure S3B**). We used the red color to represent the mutation types, and the blue color to represent the wild types. The diagram shows that the mutation type for each gene owns higher TMB. The result of differential analysis of TMB between the high-risk and low-risk group is shown in **Supplementary Figure S3C**. TMB in low-risk group is significantly higher than that in the high-risk group. As shown in **Supplementary Figure S3D**, the survival probability in the high-TMB group is higher than the low-TMB group. On the side, analyzing the survival probability jointly with TMB index, patients in the “high-TMB and low-risk” group have best prognosis (**Supplementary Figure S3E**). All these results bear out that high-TMB truly could be reckoned as a protective factor in SKCM patients. We observed extensive copy number variations (CNV) on fourteen key genes consisting of the groundwork for the signature through the CNV analysis. Among these genes, *HAPLN3*, *ERAP2*, *IFITM1*, *BCHE*, and *NMI* showed high CNV amplification frequency. In contrast, *KIR2DL4*, *CCL8*, *TLR2*, *JSRP1*, *TYRP1*, *GBP2*, *UBA7*, *KRT17*, and *SEMA4D* had significantly high CNV deletion frequency (**Supplementary Figure S3F**). The positions of CNV of the 14 hub genes on human chromosomes are shown in **Supplementary Figure S2G**.

### Tumor Microenvironment (TME) in SKCM Patients

**Figure 9:**
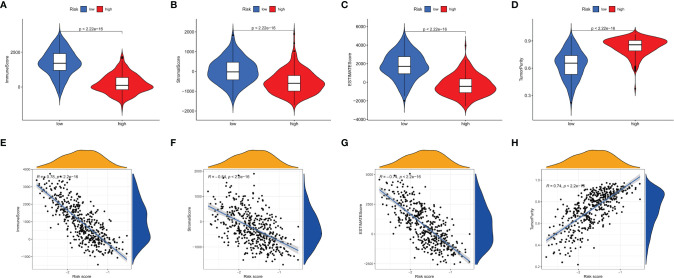
Tumor microenvironment analyses. Comparisons between high-risk group and low-risk group in terms of immune score, stromal score, ESTIMATE score **(A–C)** and tumor purity **(D)**. The relationship between the risks core and immune score, stromal score, ESTIMATE score **(E–G)** and tumor purity **(H)** in tumor tissues.

We used the ESTIMATE algorithm to calculate estimate score, immune score, stromal score, and tumor purity. Compared with the low-risk group, the immune score, stromal score and estimate score (**Figures 9A–C**) were higher in the high-risk group (p <0.001). Tumor purity (**Figure 9D**) was lower in the low-risk group. Moreover, a correlation analysis suggested risk score had a significant negative relationship with immune score, stromal score and estimate score (**Figures 9E–G**), and it had a significant postitive relationship with tumor purity (**Figure 9H**).

### Patients in the Low-Risk Group had Better Immune Function, With Higher Immune Cell Content, Expression of CYT and Immune Checkpoint Genes

**Figure 10:**
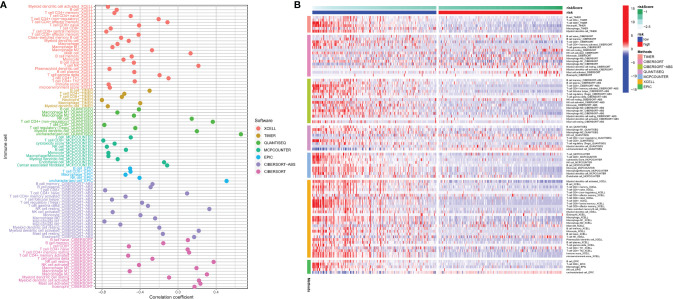
The risk score correlated with the presence of many kinds of immune cells, which was analyzed *via* XCELL, TIMER, QUANTISEQ, MCPCOUNTER, EPIC, CIBERSORT-ABS, CIBERSORT **(A)**. The heatmap shows the differential analysis of different numbers of immune cells between the high-risk and low-risk group **(B)**.

To better understand the correlation between risk score and immune cell content, the Spearman correlation analysis and Wilcoxon rank-sum test were run *via* 7 different software programs. The results are shown in **Figure 10A**. The correlation coefficient varied significantly among different types of immune cells, namely, B cells, T cells, macrophages, NK cells, neutrophils, myeloid dendritic cells, etc. Moreover, bulk differential analyzes on the amount of immune cells between the high-risk and low-risk group were also conducted *via* 7 different software programs, and the results are concordant among different software programs and reveal that the content of many immune cells differ vastly between the high-risk and low-risk group (**Figure 10B**). These results manifest that this signature has close correlation with immune, which elucidates that the signature may be an important immune marker. Furthermore, the mRNA expression landscape between the high-risk and low-risk group of a large number of immune checkpoint genes was shown in **Supplementary Figure S4A**. The differential analysis on the expression level of PD-1, PD-L1, and CTLA-4 between the high-risk and low-risk groups was performed. To underline the most widely used immune checkpoint genes, we also performed Spearman correlation analysis on PD-1, PD-L1, CTLA-4 and calculated risk scores. The expression level of the three genes is negatively correlated with the risk scores (**Supplementary Figures S4B–D**). The results showed that their expression level was higher in the low-risk group than that in the high-risk group (**Supplementary Figures S4E–G**). In addition, expression of CYT, GZMA, and PRF1 were higher in the low-risk group than high-risk group (**Supplementary Figures S5A–C**). And they were negatively correlated with risk score for SKCM patients (**Supplementary Figures S5D–F**). In **Supplementary Figures S5G–I**, we could see that CYT, GZMA and PRF1 had significant correlation with many immune cells, especially with CD8^+^ T cells (correlation coefficient >0.5, p <0.001). Results above may imply that our signature is a good reflection of CYT.

### Gene Set Enrichment Analysis

To further verify the observation based on this risk score model, Gene Set Enrichment Analysis (GSEA) was utilized to seek out enriched pathways in the KEGG and GO databases. We screened out eligible gene sets from KEGG and databases, and selected the most specific pathways. As shown in **Supplementary Figure S6A**, some gene sets were significantly upregulated in the high-risk subgroup, such as nitrogen metabolism, olfactory transduction, oxidative phosphorylation, parkinsons disease and ribosome. Some gene sets were significantly enriched in the low-risk subgroup, such as antigen processing and presentation, cell adhesion molecules cams, chemokine signaling pathway, cytokine–cytokine receptor interaction, hematopoietic cell lineage (**Supplementary Figure S6B**). In GO database, some gene sets were significantly upregulated in the high-risk subgroup, such as cornification, epidermal cell differentiation, epidermis development, keratinization, keratinocyte differentiation (**Supplementary Figure S6C**). Some gene sets were significantly enriched in the low-risk subgroup, such as activation of immune response, adaptive immune response based on somatic recombination of immune receptors built, alpha beta t cell activation, antigen processing and presentation, antigen receptor mediated signaling pathway (**Supplementary Figure S6D**). The abundant results may particularly inspire us to conduct further studies on the pathogenesis of SKCM tumor progression.

### Risk Probabilities of SKCM Patients Can be Predicted by This Signature Based on Clinical Traits

For the purpose of letting the signature better serve clinical needs, we conducted a series of analyzes on the relationship between the 14-gene signature and clinical characteristics. Differential analysis on the risk scores of subgroups with various T stage was performed. The diagram shows that along with the progression of the disease, the risk score accordingly elevates (**Supplementary Figure S7A**). Additionally, we introduced a nomogram as a measure of risk scores for SKCM patients (**Supplementary Figure S7B**). Cablibration curves (**Supplementary Figure S7C**), ROC curves (**Supplementary Figure S7D**) and DCA curves (**Supplementary Figure S7E**) were drawn to indicate the predictive accuracy of the signature.

## Discussion

The incidence of skin cutaneous melanoma (SKCM) continues to rise globally (33). SKCM is the deadliest type of skin cancer because of its early spread *via* the lymphatic vessels into lymph nodes and distant organs (34), leading to a remarkably poor prognosis and high recurrence rate. Traditional therapies have their limitations in improving the prognosis of SKCM patients. It is gratifying that the treatment landscape has shifted dramatically over a short period of time (6). Immunotherapy is reckoned as the most promising one of emerging treatments, but not all patients can benefit from it. Due to ubiquity of the immune system, immune-related adverse effects affect patients and even may lead to potentially life-threating conditions (35). Therefore, the identification of biomarkers that can predict immune responses of patients toward the specific treatment strategy so that doctors can choose the most suitable patients who will benefit from it is a prime objective of tumor study.

We noticed that in 2015, Rooney et al. elucidated the CYT value as the potential landmark that could be used to predict prognosis in cancers and had associations with counter-regulatory immune responses, which may contribute to reveal mechanisms of tumor development (18). Thus, genes associated with the CYT level are needed in order to help us better understand immune changes in human body during immunotherapy treating. It is noteworthy that in colorectal cancer, patients with higher CYT-values showed a more sensitivity to ICIs than those with lower CYT-values (19). Based on this, we identified CYT-related genes (CYTRG), established a CYT-related prognostic model, validated novel therapeutic treating targets for immunotherapies, enriched the thoughts for the treatment on SKCM in this study. For the first time, we surprisingly built a bond between SKCM and CYT score.

The CYT was calculated as the geometric mean of the GZMA and PRF1 expression in TPM. GZMA from NK cells and cytotoxic T lymphocytes (CTLs) activates gasdermin B (GSDMB) to trigger pyrotosis in target cells, which has been thought as a factor enhancing antitumor immunity (36). PRF1 also plays an important role in keeping the ability of NK cells and CTLs to strike down target cells, protecting the organism from immunosuppression and mainting immune regulation (37). Hence, through the primary analysis, we found that CYT was a protective factor for the prognosis of SKCM patients, which was within our expectations. Then, samples from TCGA database were divided into the high- and low-CYT group based on the median value of CYT scores.

Subsequently, 864 CYTRG were screened out, which further confirmed that CYT may possess abundant value in predicting prognosis for SKCM. This assumption was proved then. Fourteen CYTRG with relevant prognostic and predictive implications were identified and were used to construct the risk score model. Among them, eleven (*KIR2DL4*, *GBP2*, *SEMA4D*, *CCL8*, *UBA7*, *NMI*, *HAPLN3*, *JSRP1*, *TLR2*, *IFITM1*, and *ERAP2*) were favorable prognostic factors, whereas the other three (*BCHE*, *KRT17*, and *TYRP1*) were hazardous. Interestingly, some of them have already been verified to play an important part in SKCM. Zhou et al. (38) demonstrated that the low expression of *KIR2DL4* is significantly associated with poor prognosis in SKCM. Moreover, *KIR2DL4* as a receptor on HLA-G, has been thought as one of potential targets for immunotherapy to treat cancer (39). Fillmore et al. established stable clones constitutively expressing *NMI* (N-Myc interactor) in both breast and melanoma cell lines and eventually proved that *NMI* retards tumor growth (40). Also, Compagnone et al. (41) once gave evidence that *ERAP2* may promote immune responses mediated by T cells and NK cells to certain cancers, with low expression related to poor prognosis. In consequence, the established signature can provide novel biomarkers for further studies. It could offer ideas for us to assess prognosis of SKCM patients and we found that in the low-risk group, DSS for SKCM patients was indeed longer than that in the high-risk group.

Whereafter, the close relationship between the DSS and CYT and other clinical features was also determined. Moreover, we verified the independence of this 14-gene signature as a prognosis predictor. Besides, a nomogram was built to visualize our model. Nomograms are widely used for cancer prognosis (42). Through multiple analyses, the signature was believed to own a fulfilling distinctness, sensibility and authenticity.

To illustrate that the model is pragmatic in nature on guiding clinical drug use, firstly we found that the expression levels of gene in this signature were expressively correlated with the sensitivity of various kinds of drug in the CellMiner database, which integrates the NCI-60 cell line database and drugs approved by the U.S. Food and Drug Administration, thought as an efficient tool to easily identify drugs that are effective against different types of cancer (43). Next we calculated IC50 to determine chemotherapeutic responses for each SKCM patient. Sorafenib and imatinib elicited a better potency in the high-risk group, while gefitinib did considerably better in the low-risk group. Sorafenib was experimented to prolong OS in mice by inhibiting migration and invasion of melanoma cells and the authors speculated it to be of potential use for treating SKCM (44). As a monotherapy or in combination with chemotherapy, sorafenib is of limited use, hence it is vital to explore biomarkers to choose the suitable patients that are more likely to respond to sorafenib (45). Likewise, as tyrosine kinase inhibitor, imatinib can regulate tumor immunity by depleting effector regulatory T cells (46), and it is gradually studied too (47–49). Gefitinib has also been explored (50, 51). Thus, this possibly could be used as reference for patients with different estimated prognosis *via* our model to choose suitable drugs. Moreover, we investigated various chemotherapeutic drugs. Gemcitabine, ZM.447439 (Aurora kinase inhibitor), NVP.BEZ235 (PI3K inhibitor), Roscovitine, NVP.TAE684 and vinblastine were more sensitive to patients in the low-risk group, while vinorelbine, docetaxel, doxorubicin were more sensitive to patients in the high-risk group. Chemotherapy always has a major role to play among all traditional therapies (5), therefore the findings in our study can be applied for guiding clinical chemotherapy in patients with SKCM.

Through a series of rigorous screening, our model identified that mRNA expression levels of 14 hub genes had differences between the normal/tumor group, and between the high-/low-risk groups. Besides, nine hub genes had differences at the protein expression levels between the normal/tumor tissues. In the further analysis of the 14 hub genes, *IFITM1*, *UBA7*, *CCL8*, *HAPLN3* and *SEMA4D* emerged as the most important ones for the prognosis in SKCM patients. Among all listed genes, *GBP2*, *TYRP1* and *IFITM*1 are of intense interest to further discussion. Guanylate binding protein 2 (*GBP2*) belongs to the vast guanine-binding protein (GBP) family that is consumingly induced by interferon- γ (IFN-γ). Its role in tumorigenesis has received increasing attention in recent years. Notably, Ji et al. (52) demonstrated that *GBP2* reinforces anti-tumor functions by intercepting the Wnt/β-catenin pathway in SKCM and enhances prognosis. Yu et al. (53) found that *GBP2* promotes glioblastoma invasion through Stat3/fibronectin pathway. While in breast cancer, *GBP2* can also be stated as a tumor suppressor gene according to experimental evidence of scientists (54, 55). Sadly, there lacks solid studies on functions of GBP2 in SKCM formation for now, which also gives preliminary inspirations. On the contrary, human tyrosinase related protein 1 (*TYRP1*) is a melanosome protein involved in the pigmentary machinery of melanocytes and well-studied for its emerging roles in the malignant melanocyte and melanoma progression (56). Gilot et al. (57) even explored in depth that a reduction in the *TYRP1* mRNA level should restore the tumor-suppressor activity of miR-16 and highlighted miRNA displacement as a promising targeted therapeutic approach for melanoma. The family of interferon-induced transmembrane (IFITM) proteins is interferon induced antiviral proteins, localized in the plasma and endolysosomal membranes. With regard to IFITM1, also known as 9-27 or Leu13, is reported to be overexpressed in a wide range of neoplasms and thought as an independent prognostic biomarker for patients with certain tumor types (58). Its role in SKCM prgression stays relatively obscure. Yang et al. (59) used to speculate that *IFITM1* functions as a tumor suppressor gene and arrived at a preliminary confirmation of its prognostic role for SKCM. These results support that our model is of great value in predicting prognosis for SKCM patients, and hub genes in the model are potentially important from both a fundamental and practical point of view.

Tumor mutation burden (TMB) refers to the number of gene mutations within tumors. Considering its close connections with immune checkpoint inhibitor (ICI) treatments and other immunotherapies, high-TMB has been focused on its useful role as a novel biomarker for planning treatments and selecting ICIs across some cancer types, melanoma included (60–62). High TMB might promote neoantigen generation and T cells can react to neoepitopes generated from mutated genes that bind to MHC molecules, causing effective antitumor immune response (63). Chalmers et al. (64) analyzed 100,000 human cancer genomes and arrived at a conclusion that a substantial part of cancer patients with high TMB may benefit from immunotherapy. High TMB is associated with better prognosis in patients receiving ICI treatment (65). Herein we analyzed the somatic mutation profiles in SKCM samples. A landscape on mutation types of fourteen key genes in our model was shown. A series of results through the mutation analysis told us that high-TMB was connected with lower risk scores in SKCM patients and patients with higher TMB had better survival. Firstly, our results convey the conclusions that high-TMB in SKCM patients may equal to longer lifespan. Secondly, this might give thoughts for guiding ICI treatment for SKCM patients.

Furthermore, we analyzed tumor microenvironment (TME) by using the ESTIMATE algorithm. TME serves as a nutrient sink on which the tumor cells feed and develop (66). Groundbreaking studies in melanoma, ovarian and colorectal cancer have shown that certain features of the TME—in particular, the degree of tumor infiltration by cytotoxic T cells—can predict a clinical outcome of a patient (67). The classical tool—ESTIMATE computational method was used to estimate the ratio of immune-stromal component in TME, viewed in the form of three sorts of scores: immune score, stromal score, and ESTIMATE score. The stromal scores ranged from −1,778.68 to 1,898.41, the immune scores ranged from −1,458.20 to 3,748.11, and the ESTIMATE scores ranged from −2,582.43 to 5,069.01. Then we found that stromal scores, immune scores and ESTIMATE scores were all lower in the high-risk group than those in the low-risk group, which meant higher TME score contributed to better prognosis for SKCM patients.

Next, we analyzed the infiltration of immune cells in patients with SKCM. Tumor-infiltrating immune cells play a significant role in regulating responses to immunotherapies. Seven common methods were used to evaluate the correlation between tumor infiltrating immune cells and risk scores, namely, XCELL (68), TIMER (69), QUANTISEQ (70), MCPCOUNTER (22), EPIC (71), CIBERSORT-ABS (72), and CIBERSORT (73). We found that significant relation existed between risk scores and different types of immune cells, such as B cells, T cells, macrophages, NK cells, neutrophils, and myeloid dendritic cells. B cells are considered to be the main effector cells of humoral immunity which inhibit neoplastic progression by secreting immunoglobulins, promoting T cell response, and killing cancer cells directly (74). B cells are also discussed as an important prognostic and predictive biomarker in SKCM (75). Selitsky et al. (76) once experimentally confirmed that B cells can modulate the anti-tumor immune response by mediating proliferation and functional polarization of T cells, and they also found that a potential law in patients receiving CTLA-4 inhibitors where a lack of B cell response is possibly a sign of poor response to ICIs. Moreover, CD8^+^ and CD4^+^ T cells have been generally recognized as important anti-tumor immune cell subgroups with their cancer-cell killing efficacy, working as a crucial autoimmune gateway against cancer intrusion of an organism. We also found that in the low-risk group, immune checkpoint genes were higher and so as to Treg cells, which in our view was according to the better immune function compared to the high-risk group. Previous studies have shown that the upregulation of PD-L1 and its connection to antigen-specific CD8^+^ T cells can explain the confined host immunity in cancers (known as adaptive immune resistance), yet the high expression of PD-1, PD-L1 and other immunosuppressive molecules could be attributed to not only the mutations of tumor cells, but also the induction of tumor-infiltrating cells (12, 77). In TME, higher expression of immunosuppressive molecules can represent stronger immune attack, which can benefit the patients. Low levels of immunosuppressive molecules usually mean that the tumor cells are not recognized by the immune system or the immune system is already in ruins, which to some extent explains why immune checkpoint genes universally express more in the low-risk group. Moreover, we noticed patients in the low-risk group had higher TMB value and prolonged survival than the high-risk group. This also indicated that in the low-risk group, they had better immune functions, because tumor cells should withstand the anti-tumor immunity of the body with continuous mutations and produce more immunosuppressive molecules (termed as immune escape) (78). On the contrary, low TMB may signify a rather powerful invasion of tumor cells or an extremely damaged immune system, by which tumor cells do not need mutations to tolerate tumor immunity. These speculations are consistent with the higher levels of immune infiltrating cells in the low-risk group for SKCM. In further studies, we found that CYT, GZMA and PRF1 were highly expressed in the low-risk group, significantly negatively correlated with risk scores, and expressively positively related with CD8^+^ T cell content. Thus we hypothesized that high CYT in SKCM could mediate tumor immunity through CD8^+^ T cell and lead to better outcomes. And there was a moderate positive correlation between CYT and Macrophages 1 (M1), and a moderate negative correlation between CYT and Macrophages 2 (M2). M1 is mainly involved in inflammatory responses and anti-tumor processes, while M2 shows tumor-promoting activity (79). Thus we could better assume that SKCM with higher CYT would have better clinical prognosis because of stronger mmunogenicity and a more favorable TME. Furthermore, GZMA was a potent adjuvant that induced antigen-specific cytotoxic CTLs to play a prominent part in antitumor activity in mice when co-administered with antigen (80). Inoue et al. indicated that more expression levels of PD-1 ligands, GZMA and HLA-A in melanoma tissues may be conductive to respond preferentially to nivolumab treatment by expanding oligoclonal tumor-infiltrating lymphocytes (81). PRF1 was also confirmed to have close relation with better OS by modulating tumor immunity in cancers like head and neck squamous cell carcinoma, ovarian cancer and basal-like breast tumors, and liver cancer (82–84). In summary, our findings show that the patients in the low-risk group had better survival, and provide a theoretical basis for studying pathogeniss and treatment methods of SKCM. CYT, as a protective factor in SKCM, was again confirmed.

Through the GSEA of biological pathways for different risk subgroups in different databases, we found that a diverse array of immune-related signaling pathways showed significant differences, which lies within our expectations. Interestingly, the pathways like activation of immune response, antigen processing and presentation, cell adhesion molecules (CAMs) were significantly downregulated among high-risk group. Antigen processing and presentation is a classic adaptive immune-response course in which dendritic cells (DCs) are considered to play a central role potently and professionally (85). In many tumors, an immunosuppressive microenvironment can be attributed to the dysfunction of DCs to recognize, process, and present tumor antigens to T cells (86). The loss of CAMs in the early stage of melanoma allows the tumor cells to proliferate and intrude the dermis with the reduction of anchorage on the basement membrane and between the ambient keratinocytes (87), which allows distant metastasis in the follow-up mutations. These results illustrate that CYT regulates tumor pathogenesis by modulating various immune responses. Remarkably, our GSEA also offers some new insights into tumor mechanism governing, many of them certainly seem like an untapped area to explore. Parkinsons disease was enriched in the high-risk group. Forés-Martos et al. (88) demonstrated that significant genetic correlations exist between Parkinson’s disease, prostate cancer, and melanoma.

As is mentioned above, within this study, we found that PD-1, CTLA4 and PD-L1 genes were expressed more in the low-risk group. PD-1, CTLA4, PD-L1 inhibitors currently are among the hottest ICIs, contribute much to treat cancers, including SKCM. It may roughly possess accurate predictive capacity to identify patients who could respond well to immunotherapies. The underlying mechanism for this may be ascribed to that the higher TMB in the low-risk group contributes to more neoantigens generated by tumor mutations and more T lymphocytes infiltrated by tumors, which makes the tumor more immunogenic along with a stronger anti-tumor immune response [15]. In fact, this is consistent with the result that SKCM patients with higher TMB expression have better outcomes. However, it is cautionary to note that our results suggest that immune checkpoints are generally upregulated in SKCM patients, noting that they are more prone to immune escape during immunotherapy. These conclusions offer practice guidance, and shed a new light on the immunotherapy for SKCM.

Nevertheless, there were limitations in this study. This was a retrospective study with datasets from the TCGA database, lacking specific clinical information such as treatment and recurrence records. And our conclusions need to be validated *in vivo* or *in vitro* experiments to further examine the function of CYTRGs in SKCM progression and to understand mechanisms of neoplasia better. Still, prospective clinical studies are welcome to verify phenomena reflected in this research.

In summary, our analyses of gene expression matrix and corresponding clinical characteristics identified 14 prognosis-related CYTRGs in skin cutaneous melanoma. Based on the clinical characteristics of CYT, we constructed a novel risk scoring model, which can effectively evaluate the prognosis for SKCM patients and forecast the benefit of SKCM immunotherapy. Our study illustrated that CYT may positively influence the development and outcome of tumors by modulating tumor microenvironment. Thus, poor prognosis of high-risk patients with SKCM may be attributed to the lower immune functions of immune cells. And different sensitivity to therapeutic drugs between the high- and low-risk groups could also be due to differential expressions of immune checkpoints and cytokines. Significantly, our study showed that low-risk patients with SKCM benefit more from immunotherapies and the model can be employed as a key tool to facilitate rational drug use and guide clinical treatment.

## Conclusion

Our study is the first to establish a 14 CYT-related-gene prognostic model. Abundant analyzes verify that this signature can be used as a promising predictive biomarker and therapeutic target for SKCM patients.

## Data Availability Statement

The datasets presented in this study can be found in online repositories. The names of the repository/repositories and accession number(s) can be found in the article/**Supplementary Material**.

## Author Contributions

HZ is responsible for writing and submitting the papers. YL is responsible for data collection and analysis and the production of pictures. DH and SL are responsible for the ideas and guidance. All authors listed have made a substantial, direct, and intellectual contribution to the work and approved it for publication.

## Conflict of Interest

The authors declare that the research was conducted in the absence of any commercial or financial relationships that could be construed as a potential conflict of interest.

## Publisher’s Note

All claims expressed in this article are solely those of the authors and do not necessarily represent those of their affiliated organizations, or those of the publisher, the editors and the reviewers. Any product that may be evaluated in this article, or claim that may be made by its manufacturer, is not guaranteed or endorsed by the publisher.

## Supplementary Material

Supplementary Figure 1 Using CellMiner method to conduct correlation analysis between drugs and targeted genes.

Supplementary Figure 2 The levels of gene in the signature correlate with CYT in SKCM patients.

Supplementary Figure 3 The landscape of mutations of somatic cells in SKCM patients. **(A)** Waterfall map shows the mutational conditions of 14 genes involved in building the signature. **(B)** Boxplot displays the TMB difference of SKCM patients in TCGA-cohort. The red represents the mutation types, and the blue represents the wild types. *P < 0.05; **P < 0.01; ***P < 0.001. **(C)** Differential analysis of TMB between the high-risk and low-risk group. **(D)** Survival analysis between the high-TMB and low-TMB group. **(E)** Survival anlaysis on the 14-gene signature with the combination of TMB. The copy number variation (CNV) frequency percentage of the fourteen hub genes in SKCM. The red dot represents the CNV amplification, and the green dot represents the CNV deletion **(F)**. The location of CNV of 14 hub genes on human chromosomes **(G)**.

Supplementary Figure 4 Correlation analysis and differential analysis of immune checkpoint genes. The overview of differential expression of immune checkpoint genes **(A)**. Correlation analysis and differential analysis of PDCD1 **(B, E)**, CTLA4 **(C, F)**, and CD274 **(D, G)**.

Supplementary Figure 5 Correlation analysis and differential analysis of CYT, GZMA and PRF1. Comparations between high-risk group and low-risk group in terms of CYT, GZMA, PRF1 expression **(A–C)**. The relationship between the risks core and CYT, GZMA, PRF1 expression **(D–F)** and their correlation coefficients **(G–I)**.

Supplementary Figure 6 Gene set enrichment analysis. Representative enrichment plots generated in the KEGG database, the pathways enriched in the high-risk group **(A)** and low-risk group **(B)** are displayed. Representative enrichment plots generated in the GO database, the pathways enriched in the high-risk group **(C)** and low-risk group **(D)** are displayed.

Supplementary Figure 7 Correlation analysis on the relationship between the 14-gene signature and clinical characteristics. Differential analysis on risk scores of subgroups with various T stage **(A)**. The nomogram based on OS/follow-up time, tumor status/T stage to evaluate risk scores **(B)**. Cablibration curves **(C)**, ROC curves **(D)**, DCA curves **(E)** indicate the accuracy of the signature.

## Abbreviations

SKCM, skin cutaneous melanoma; CYT, cytolytic activity; TCGA, The Cancer Genome Atlas; GEO, Gene Expression Omnibus; DEG, differentially expressed genes; NMF, non-negative matrix factorization; TMB, tumor mutation burden; CNV, copy number variation; TME, tumor microenvironment; GSEA, gene set enrichment analysis; ICIs, immune checkpoint inhibitors; CTLA-4, cytotoxic T-lymphocyte-associated protein 4; PD-1, programmed cell death-1; PD-L1, programmed cell death ligand-1; GZMA, granzyme A; PRF1, perforin; OS, overall survival; DSS, disease specific survival; PFS. progression-free-survival; CYTRG, CYT-related gene; GSVA, gene set variation analysis; ESTIMATE, estimation of stromal and immune cells in malignant tumor tissues using expression data; MCP-counter, microenvironment cell populations-counter; TNM, tumor-node-metastasis; ROC, receiver operator characteristic curve; CCM, calibration curve method; PCA, principal component analysis; DCA, decision curve analysis; C-index, concordance index; RMS, restricted mean survival; IC50, half maximal inhibitory concentration; GDSC, genomics of drug sensitivity in cancer; IPS, immunophenoscore; TCIA, the cancer immunome database; HPA, human protein atlas; MAF, mutation annotation format; HRs, hazard ratios; CIs, confidence intervals; AUC, area under curve; IHC, immunohistochemical; NMI, N-Myc interactor; GBP, guanine-binding protein; IFN-γ, interferon- γ; TYRP1, tyrosinase related protein 1; IFITM, interferon-induced transmembrane; CAMs, cell adhesion molecules; DCs, dendritic cells.
