# Supplementary figures and images for: Identification of Novel Molecular Therapeutic Targets and Their Potential Prognostic Biomarkers Based on Cytolytic Activity in Skin Cutaneous Melanoma
Source: Front Oncol. 2022 Mar 8;12:844666. doi: 10.3389/fonc.2022.844666 (PMC8957259; doi:10.3389/fonc.2022.844666)

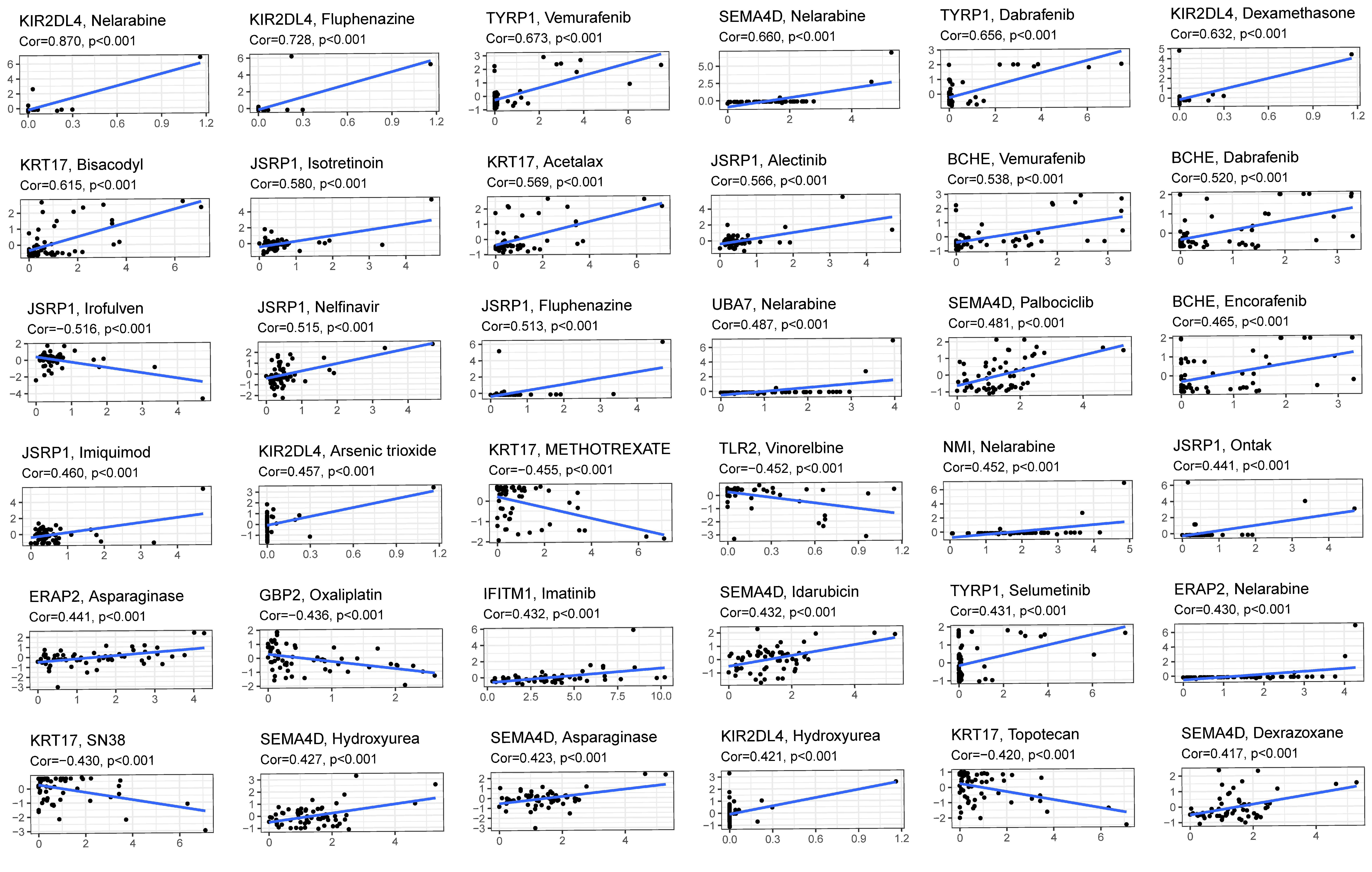

Supplement: Supplementary Figure 1 — Using CellMiner method to conduct correlation analysis between drugs and targeted genes. [file Image_1.jpeg]

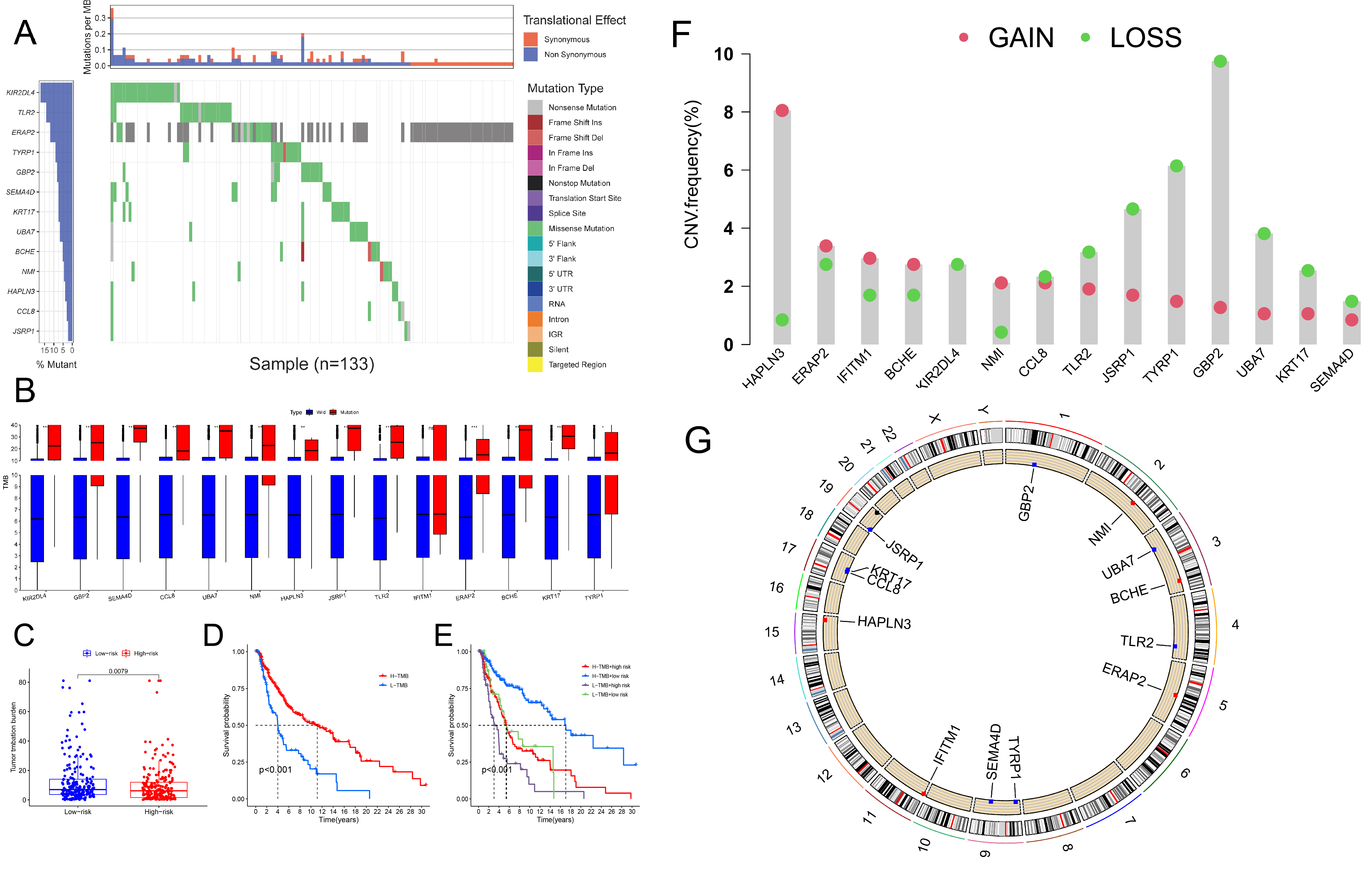

Supplement: Supplementary Figure 3 — The landscape of mutations of somatic cells in SKCM patients. (A) Waterfall map shows the mutational conditions of 14 genes involved in building the signature. (B) Boxplot displays the TMB difference of SKCM patients in TCGA-cohort. The red represents the mutation types, and the blue represents the wild types. *P < 0.05; **P < 0.01; ***P < 0.001. (C) Differential analysis of TMB between the high-risk and low-risk group. (D) Survival analysis between the high-TMB and low-TMB group. (E) Survival anlaysis on the 14-gene signature with the combination of TMB. The copy number variation (CNV) frequency percentage of the fourteen hub genes in SKCM. The red dot represents the CNV amplification, and the green dot represents the CNV deletion (F). The location of CNV of 14 hub genes on human chromosomes (G). [file Image_3.jpeg]

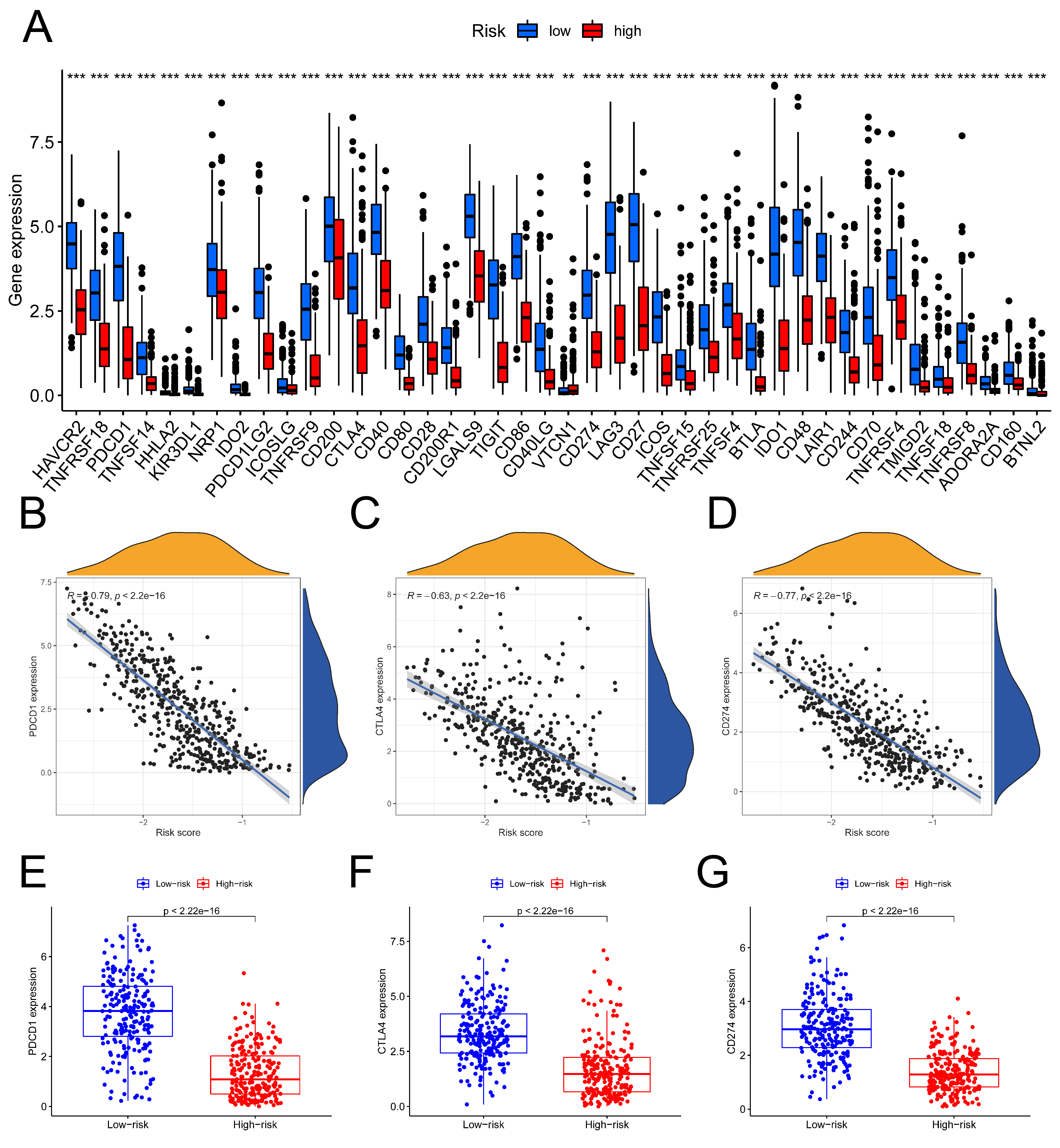

Supplement: Supplementary Figure 4 — Correlation analysis and differential analysis of immune checkpoint genes. The overview of differential expression of immune checkpoint genes (A). Correlation analysis and differential analysis of PDCD1 (B, E), CTLA4 (C, F), and CD274 (D, G). [file Image_4.jpeg]

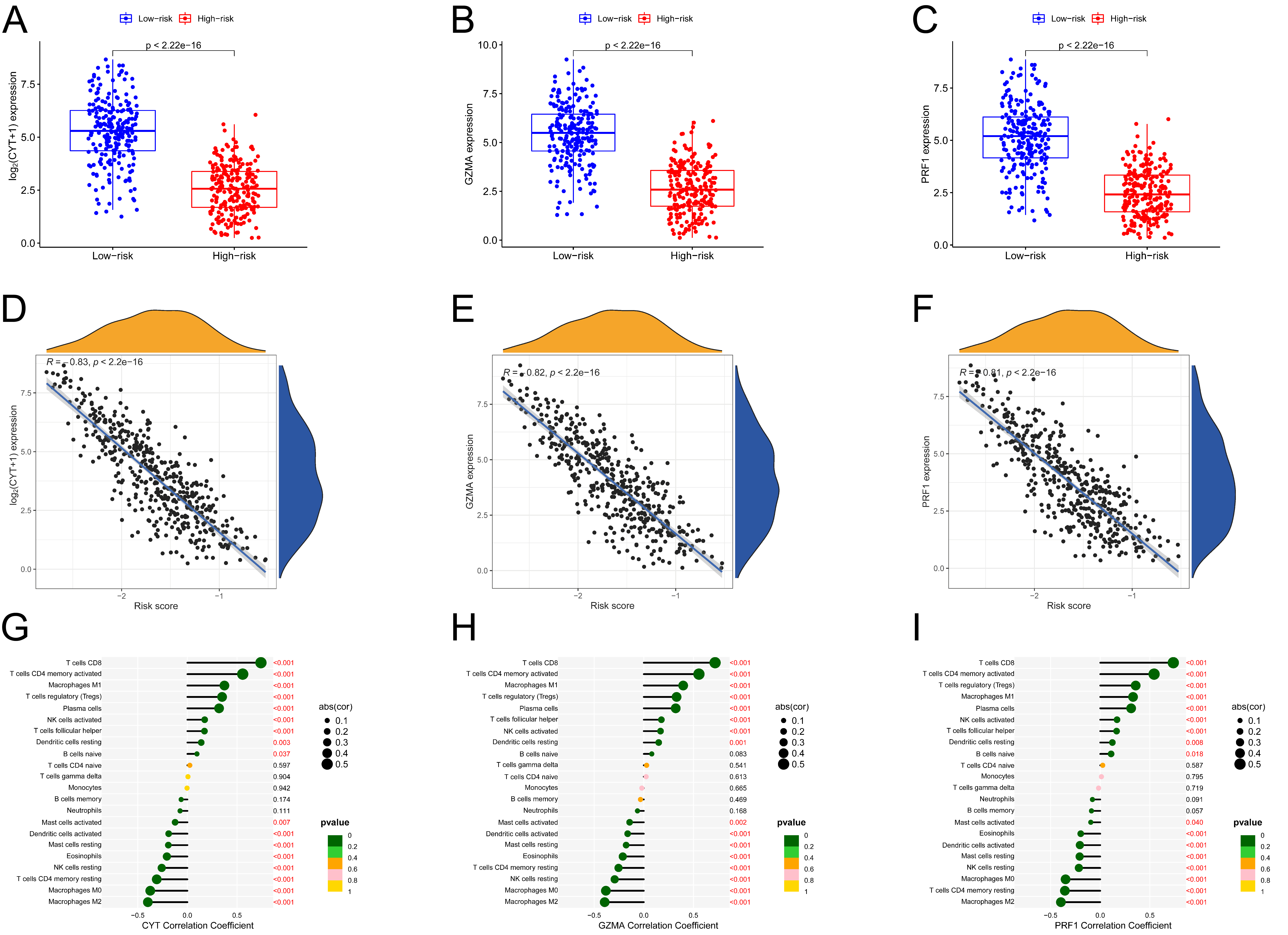

Supplement: Supplementary Figure 5 — Correlation analysis and differential analysis of CYT, GZMA and PRF1. Comparations between high-risk group and low-risk group in terms of CYT, GZMA, PRF1 expression (A–C). The relationship between the risks core and CYT, GZMA, PRF1 expression (D–F) and their correlation coefficients (G–I). [file Image_5.jpeg]

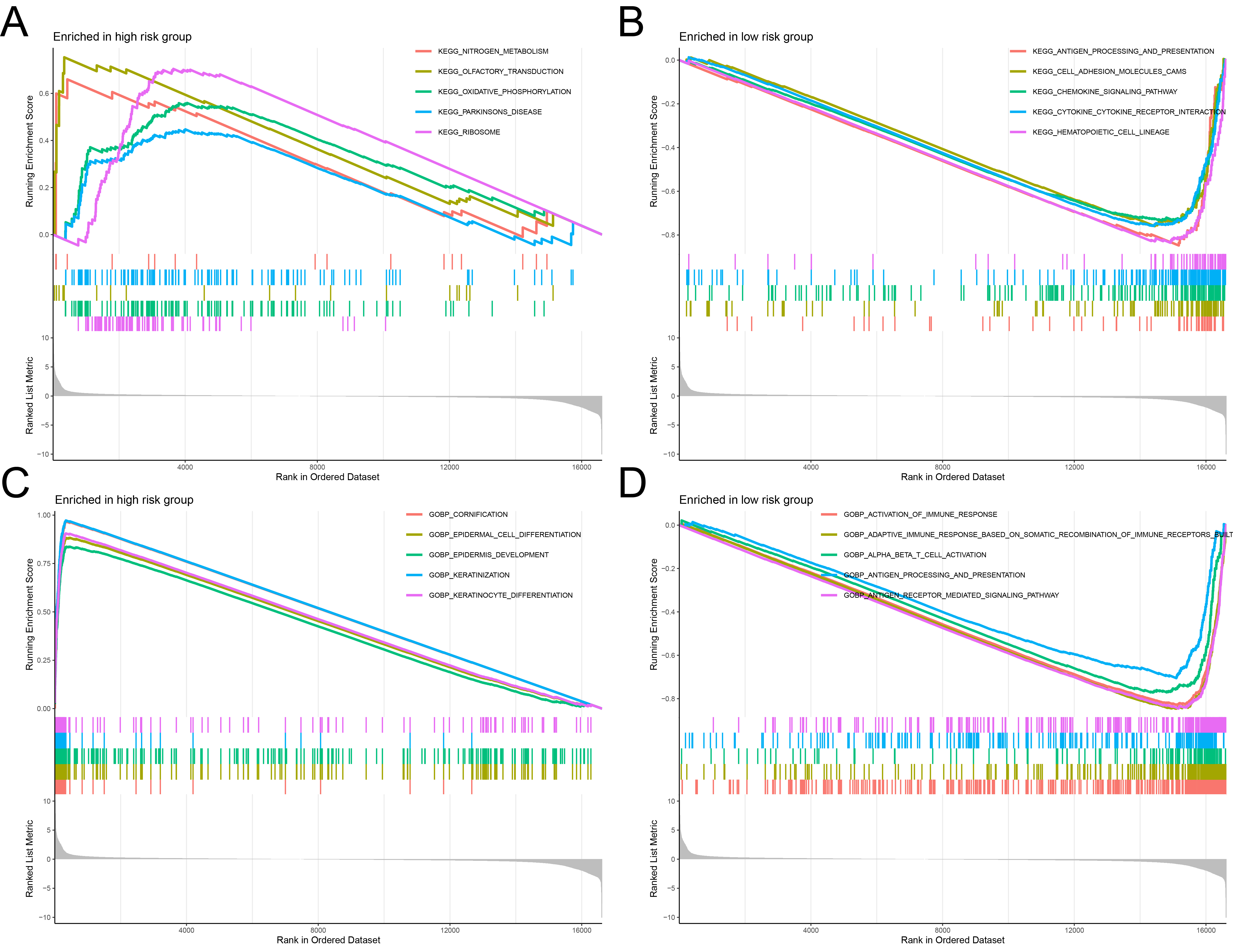

Supplement: Supplementary Figure 6 — Gene set enrichment analysis. Representative enrichment plots generated in the KEGG database, the pathways enriched in the high-risk group (A) and low-risk group (B) are displayed. Representative enrichment plots generated in the GO database, the pathways enriched in the high-risk group (C) and low-risk group (D) are displayed. [file Image_6.jpeg]

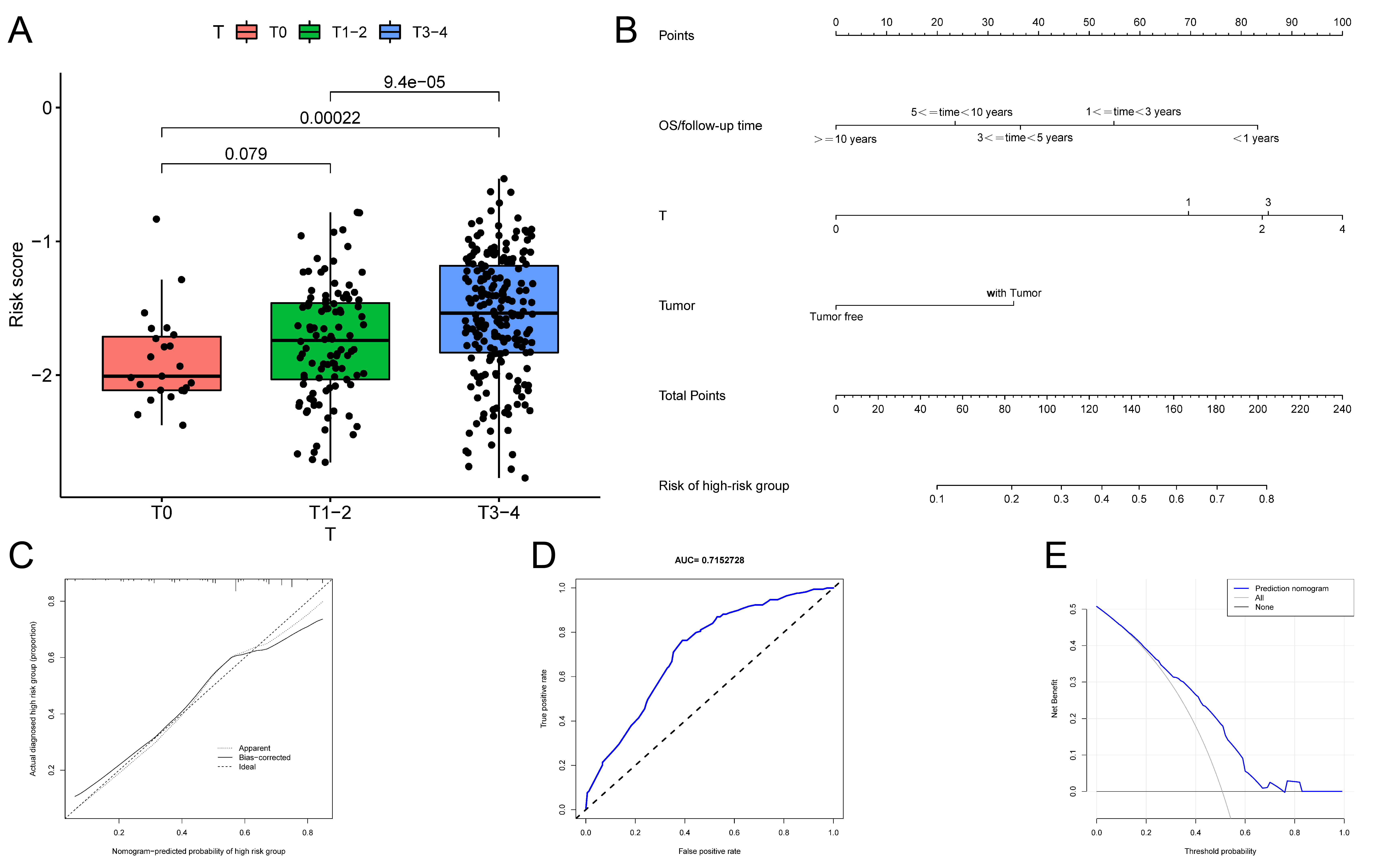

Supplement: Supplementary Figure 7 — Correlation analysis on the relationship between the 14-gene signature and clinical characteristics. Differential analysis on risk scores of subgroups with various T stage (A). The nomogram based on OS/follow-up time, tumor status/T stage to evaluate risk scores (B). Cablibration curves (C), ROC curves (D), DCA curves (E) indicate the accuracy of the signature. [file Image_7.jpeg]
